# Supplementary material for: Effects of the Water Extract of Fermented Rice Bran on Liver Damage and Intestinal Injury in Aged Rats with High-Fat Diet Feeding
Source: Plants (Basel). 2022 Feb 24;11(5):607. doi: 10.3390/plants11050607 (PMC8912322; doi:10.3390/plants11050607)
Supplement: Supplementary file 1 [file plants-11-00607-s001.zip › plants-1596336-supplementary.pdf]

Analysis of FRB components

Liquid chromatographic/mass spectrometric (LC/MS) analysis

The LC-MS analysis was performed on a Waters Acquity UPLC I-Class system and Vion IMF Quadrupole Time of Flight (QTof) Mass Spectrophotometer. The system was equipped with a Waters BEH C18 Acquity analytical column (75 μm × 150 mm, 1.8 μm). The column oven temperature was set to 40 °C, and the temperature of the auto-sampler was set to 4 °C. For each LC-HDMSE run, approximately 2 μL of sample (1 mg/mL) was loaded into the column through a 20-μL sample-loop using 98% of mobile phase A (0.1% formic acid in H2O) at a flow rate of 0.4 mL/min with a gradient elution consisting of an increase from 20% to 46% of mobile phase B (0.1% formic acid in acetonitrile (ACN)) over 25 min, and a re-equilibration step at 20% of mobile phase B for 10 min. The lock mass, 200 fmol/μL of [Leu] solution prepared with 0.1% formic acid in 30% ACN, was delivered from the auxiliary pump at a flow rate of 0.2 μL/min to the reference sprayer of the LockSpray source.

LC-HDMSE data were acquired in resolution mode with the UNIFI Scientific Information System. MS was operated in resolution mode with a typical resolving power of at least 40,000 FWHM at m/z 500. All analyses were performed using positive mode electrospray ionization (ESI) using a LockSpray source. The lock mass channel was sampled every 60 s. MS was calibrated with a Leu solution (200 fmol/μL) delivered through the reference sprayer of the LockSpray source. Accurate mass LC-HDMSE data were acquired in alternating, low energy (MS) and high energy (MSE) modes of acquisition with a mass scan range of from m/z 50 to 1000, using a capillary voltage of 2 kV, a source temperature of 150 °C, and a cone voltage of 30 V. The mass measured accuracy was <5 ppm when the instrument was operated with the lock mass, and the accuracy was the same between the low-energy and high-energy MS scans. The spectral acquisition time in each mode was 1.0 s with a 0.1-s inter-scan delay. In the low-energy HDMS mode, data were collected at a constant collision energy of 2 eV in both the trap cell and transfer cell. In the high-energy HDMSE mode, the collision energy was ramped up from 15 to 30 eV in the transfer cell only.

Nutrition analysis

The nutritional ingredients of FRB were measured by Société Générale de Surveillance (SGS) Food Laboratory (SGS, Kaohsiung, Taiwan) according to instructions of the Association of Official Analytical Communities (AOAC), Chinese National Standards (CNS) and Taiwan Ministry of Health and Welfare (MOHW). Test items included energy, protein and amino acid composition, fat (saturated fatty acids (FAs), trans FAs and FA composition), carbohydrate (sugars and fibers), moisture, ash, sodium, etc.

Supplementary Table S1. LC/MS analysis of the water extract of fermented rice bran (FRB)

Component name	Response	Mass error (ppm)	Observed m/z	Observed RT (min)
Gramisterol	871446	-0.2	Identified	413.3777
Momor-cerebroside I	570028	0.2	Identified	844.6874
24-Ethyl cholesterol	220305	-3.7	Identified	415.3919
Aristolone	91060	0.5	Identified	219.1745
Siraitic acid C	78278	-4.4	Identified	441.298
Cyclolaudenone	69508	0.2	Identified	439.3935
(24S)-5α,8α-Epidioxy-ergost-6-en-3β-ol	52815	0.4	Identified	431.3522
Phosphatidyl ethanolamines	51017	-4.3	Identified	746.5663
Lotaustralin	46075	1.8	Identified	262.129
Heterodendrin	46075	1.8	Identified	262.129
Kulactone	42421	-4.4	Identified	453.3343
Vitamin B5	41627	1.5	Identified	220.1183
Parkeol	39478	-1.5	Identified	427.3928
Δ7-Stigmasterol	36768	0.8	Identified	411.3625
(E)-Hexadecyl-ferulate	36420	0.5	Identified	419.3158
Deoxycholic acid	32860	-5.3	Identified	393.2979
Aristolone	32318	1.5	Identified	219.1747
Chrysotoxine	28741	-7.7	Identified	319.1516
Patuletin-7-O-[6''-(2-methylbutyryl)]-glucoside	28284	1	Identified	565.1558
Phosphatidyl ethanolamines	25646	-4.2	Identified	746.5663
2-Monolinolein	23816	-2	Identified	355.2836
Ocotillol acetate	21532	0.7	Identified	503.4099
Chebuloside Ⅱ	20322	-1.6	Identified	667.4041
o-Coumaric acid	19937	2.5	Identified	165.055
Folinic acid	18438	0.6	Identified	474.1734
Bis(2-ethylhexyl) phthalate	17776	2.8	Identified	391.2854
Methyl cinnamate	16213	1	Identified	163.0755
Phthalic anhydride	15948	2	Identified	149.0236
Nicotinic acid	15792	1.1	Identified	124.0394
Apigenin-7-O-β-D-glucuronopyranoside	15566	1	Identified	445.1134
Dulcitol	15262	0.4	Identified	183.0864
Alisol A	14498	1.4	Identified	491.3738
Deoxycholic acid	14202	-5.3	Identified	393.2979
Pantothenic acid	13723	1.4	Identified	248.1496
Trichosanic acid	13648	1.2	Identified	279.2322
Prunasine	12514	-0.3	Identified	296.1128
4-Feruloylquinic acid	12237	-0.9	Identified	369.1177
p-Tolualdehyde	11985	2.4	Identified	123.0444
Stearidonic acid	11732	0.3	Identified	277.2163
Pachymic acid	11257	2.1	Identified	529.3899
Stigmastan-3,6-dione	10550	-1	Identified	429.3723
Patuletin-7-O-[6''-(2-methylbutyryl)]-glucoside	10278	-0.2	Identified	565.1551
(-)-Epiafzelechin-3-O-β-D-allopyranoside	10202	-7.1	Identified	437.1411
Cynatratoside B	9840	5.2	Identified	779.4253
n-Butyl benzoate	9584	2.2	Identified	179.107
Cynatratoside B	9353	5.5	Identified	779.4255
9,16-Dioxyhydroxy-10,12,14-triene-18 carbonic acid	9059	-5.1	Identified	311.2201
Gigantol	9033	-8.1	Identified	261.11
Mahuannin G	8736	8.2	Identified	543.133

Glycocholic acid	8588	0	Identified	466.3163
Glycodeoxycholic acid	8450	-9.6	Identified	450.3171
2-Hydroxybenzyl-3-hydroxybenzoate	8195	-9.4	Identified	245.0785
Thannilignan	8191	-3	Identified	331.153
Parkeol	7935	-2.7	Identified	427.3923
Methyl cinnamate	7838	1.5	Identified	163.0756
(22E,24R)-5α,8α-Epidioxyergosta-6,9,22-trien-3β-ol	7746	-8.7	Identified	427.3169
Siraitic acid C	7727	-4.8	Identified	441.2978
Stearidonic acid	7571	0.6	Identified	277.2164
Alisol F	7256	2.1	Identified	489.3585
2-Amino-3-(3,4-dihydroxyphenyl)-propanoic acid ethyl ester	7201	2.3	Identified	226.1079
Alisol I	7189	-3.6	Identified	455.3503
Kuraridinol	7189	-8.6	Identified	457.2182
(3R,4S)-4',7-Dimethoxy-3'-deoxysappanol	7188	7.3	Identified	317.1407
Nobilin C	7180	-9	Identified	335.1459
Terrestriamide	7120	8.1	Identified	328.1206
Periplocoside O	7110	8	Identified	649.3998
(-)-Epiafzelechin-3-O-β-D-allopyranoside	7065	-7.9	Identified	437.1408
Diisobutyl phatel (DIBP)	6993	0.7	Identified	279.1593
N-Methylanthranilic acid	6976	2.7	Identified	152.071
2-Amino-benzoic acid	6912	2.4	Identified	138.0553
Cycloartanol	6862	-5.1	Identified	429.4069
2-Amino-3-(3,4-dihydroxyphenyl)-propanoic acid methyl ester	6716	2.7	Identified	212.0923
Luteolin-7-O-[β-D-apiofuranosyl(1→6)]β-D-glucopyranoside	6620	1.2	Identified	581.1508
Stigmast-4-ene-3,6-dione	6515	-2.9	Identified	427.3558
Stearidonic acid	6454	0.5	Identified	277.2163
9,16-Dioxyhydroxy-10,12,14-triene-18 carbonic acid	6444	-5.8	Identified	311.2199
Tenacigenin C	6394	2.1	Identified	383.2436
Chrysotoxine	6355	-7	Identified	319.1518
24-Methylenelanost-8-en-3β-ol	6316	-9.8	Identified	441.4048
9,16-Dioxyhydroxy-10,12,14-triene-18 carbonic acid	6287	-2.9	Identified	311.2208
9,16-Dioxyhydroxy-10,12,14-triene-18 carbonic acid	6276	-6.6	Identified	311.2196
16-Oxo alisol A	6230	-4.3	Identified	505.3502
Diosgenin acetate	6159	-5.6	Identified	457.3287
Atratoside A	6115	-5.7	Identified	777.4375
Stearidonic acid	6046	-1.5	Identified	277.2158
Daturametelin D	6028	-4.7	Identified	437.2666
Delamide	5960	4.5	Identified	265.1195
Ethyl linoleate	5941	2.2	Identified	309.2795
Hydroginkgolinic acid	5902	1.6	Identified	335.2586
(E)-Ferulic acid hexacosyl ester	5848	3.2	Identified	559.4739
Coronaric acid	5823	-4.7	Identified	297.241
L-Noradrenaline	5788	3.4	Identified	170.0818
9,16-Dioxyhydroxy-10,12,14-triene-18 carbonic acid	5737	-4.1	Identified	311.2204
6-Gingerdione	5679	-0.2	Identified	293.1747
9,16-Dioxyhydroxy-10,12,14-triene-18 carbonic acid	5629	-5	Identified	311.2201
(3-Methoxycarbonylamino-4-methylphenyl)-carbamic acid methyl ester	5619	0.1	Identified	239.1026
Di (2-ethyl butyl) phthalate	5395	-6.6	Identified	335.2195
Neomangiferin	5389	-2.5	Identified	585.1436
16β-Methoxyalisol B 23-acetate	5388	-0.6	Identified	545.3833

RT, retention time.

Supplementary Table S2. Amino acid compositions of the water extracts of rice bran (RB) and fermented RB (FRB)

		**RB**	**FRB**
Alanine	ppm (mg/kg)	7090	8120
Arginine	ppm (mg/kg)	8870	6100
Aspartic Acid	ppm (mg/kg)	10,800	12,100
Cystine	ppm (mg/kg)	1550	1250
Glutamic Acid	ppm (mg/kg)	16,500	16,400
Glycine	ppm (mg/kg)	6500	6550
Histidine	ppm (mg/kg)	3810	3460
Isoleucine	ppm (mg/kg)	4450	5010
Leucine	ppm (mg/kg)	8010	7460
Lysine	ppm (mg/kg)	5400	6290
Methionine	ppm (mg/kg)	1470	1300
Phenylalanine	ppm (mg/kg)	5230	4630
Proline	ppm (mg/kg)	5990	12,800
Serine	ppm (mg/kg)	5090	5720
Threonine	ppm (mg/kg)	4240	5410
Tyrosine	ppm (mg/kg)	2500	3680
Valine	ppm (mg/kg)	6750	6580
Tryptophan	ppm (mg/kg)	1390	1510
GABA	(mg/100g)	15.6	220.0

GABA, gamma-aminobutyric acid.

Supplementary Table S3. Fatty acid compositions of the water extracts of rice bran (RB) and fermented RB (FRB)

		RB	FRB
Fatty acids			
Tetradecanoic acid (14:0)	g/100 g	0.06	N.D.
Hexadecanoic acid (16:0)	g/100 g	2.79	0.98
Octadecanoic acid (18:0)	g/100 g	0.34	0.17
Eicosanoic acid (20:0)	g/100 g	0.10	N.D.
Docosanoic acid (22:0)	g/100 g	0.06	N.D.
Teracosanoic acid (24:0)	g/100 g	0.15	0.10
Fatty acids composition			
Dodecanoic acid (12:0)	%	N.D.	0.07
Tetradecanoic acid (14:0)	%	0.4	0.25
Pentadecanoic acid (15:0)	%	N.D.	0.09
Hexadecanoic acid (16:0)	%	19.4	16.3
9-Cis-hexadecanoic acid (9c-16:1)	%	0.16	0.23
Heptadecanoic acid (17:0)	%	N.D.	0.1
Octadecanoic acid (18:0)	%	2.33	3.74
9-Cis-octadecanoic acid (9c-18:1)	%	44.9	39.6
11-Cis-octadecanoic acid (11c-18:1)	%	1	1.06
9,12-Cis-octadecadienoic acid (9c, 12c-18:2)	%	27.6	31.9
9,12, 15-Cis-octadecatrienoic acid (9c, 12c, 15c-18:3)	%	0.9	2.13
9-Trans, 12-cis, 15-cis-octadecatrienoic acid (9t, 12c, 15c-18:3)	%	N.D.	0.08
6, 9, 12-Cis-octadecatrienoic acid (6c, 9c, 12c-18:3)	%	N.D.	1.03
Eicosanoic acid (20:0)	%	0.72	0.68
11-Cis-eicosanoic acid (11c-20:1)	%	0.65	0.52
Docosanoic methyl ester (22:0)	%	0.49	0.68
7, 10, 13, 16, 19-Cis-docosapentaenoic acid (7c, 10c, 13c, 16c, 19c-22:5)	%	0.18	N.D.
Tetracosanoic acid (24:0)	%	1.2	1.55

The test results “N.D.” stand for Limit of Quantification.

Supplementary Table S4. Components in the water extracts of rice bran (RB) and fermented RB (FRB)

		**RB**	**FRB**
Energy	(kcal/100g)	426.3	285.3
Protein	(g/100g)	12.9	15.5
Fat	(g/100g)	14.3	5.3
Saturated fatty acid	(g/100g)	3.50	1.26
Trans fatty acid	(g/100g)	N.D.	N.D.
Total carbohydrates	(g/100g)	61.5	43.9
Total sugars	(g/100g)	6.6	13.0
Dietary fiber	(g/100g)	19.1	6.2
Moisture	(g/100g)	4.5	14.3
Ash	(g/100g)	6.8	21.0
Sodium	(mg/100g)	5.1	72.0
Calcium	ppm (mg/kg)	506	1630
Phosphorus	ppm (mg/kg)	16,400	51,100

The test results "N.D." stand for Limit of Quantification.
